# Erratum, Vol. 1, No. 1

**Published:** 2004-06-15

The title of the article “Law as a Tool for Preventing Chronic Diseases: Expanding the Spectrum of Effective Public Health Strategies” appeared incorrectly in the article and in the suggested citation of the article as “Law as a Tool for Preventing Chronic Diseases: Expanding the Range of Effective Public Health Strategies” (Vol. 1: No. 1, January 2004). The title appeared correctly in the Table of Contents.

The information was corrected on our Web site on April 22, 2004. The corrected article appears online at http://www.cdc.gov/pcd/issues/2004/jan/03_0033.htm.

We regret any confusion this error may have caused.

